# Prevalence and factors associated with abortion and unsafe abortion in Nepal: a nationwide cross-sectional study

**DOI:** 10.1186/s12884-018-2011-y

**Published:** 2018-09-17

**Authors:** Abinath Yogi, Prakash K.C, Subas Neupane

**Affiliations:** 1 Research Triangle Institute (RTI) International, Oasis building, Patan Dhoka, Lalitpur, 401 Nepal; 2 Faculty of Social Sciences (Health Sciences), University of Tampere, FI-33014 Tampere, Finland

**Keywords:** Abortion, Unsafe abortion, Prevalence, Determinants, Nepal demographic and health survey, Demographic and health survey

## Abstract

**Background:**

Abortion is one of the leading causes of maternal death in low- and middle-income countries. In Nepal, abortion is reported to be the third leading cause of maternal death. We aimed to investigate the prevalence and factors associated with abortion and unsafe abortion in Nepal.

**Methods:**

This study is based on a nationally representative sample of the Nepal Demographic and Health Survey 2011. Women who had ever had a terminated pregnancy (*n* = 2395) were studied. The survey elicited information on the most recent abortion. Unsafe abortion was defined according to the providers of abortion services. Binary logistic regression was used to calculate odds ratios (ORs) and 95% Confidence Intervals (CIs) of abortions and unsafe abortions due to demographic, socio-economic and lifestyle-related characteristics. The interaction of the reason for abortion with age and educational status in predicting unsafe abortion was calculated using the predictive margins and their 95% CI.

**Results:**

The five-year prevalence of abortion was 21.1% among women of reproductive age who ever had a terminated pregnancy and 16.0% of total abortions were unsafe. Women of Buddhist religion (OR 2.15; 95% CI 1.04, 4.44), those who were literate (secondary level education OR 1.69; 95% CI 1.22, 2.34), those who knew about legal abortion (OR 1.88; 95% CI 1.41, 2.52) and those who were aware of safe places for abortion services (OR 4.96; 95% CI 3.04, 8.09) were more likely to undergo an abortion. Likewise, women in age group 25–34 years (OR 0.43; 95% CI 0.19, 0.97) and those who were in the richest wealth quintile (OR 0.10; 95% CI 0.04, 0.25) were less likely to undergo an unsafe abortion. Educated women of 25–34 years reporting “health risk” as the reason for abortion had a decidedly lower probability (< 10.0%) than the others of going through the unsafe abortion.

**Conclusions:**

The prevalence of abortion in Nepal remains high. Education, religion, age, knowledge about legal abortion and safe places to undergo abortion were the major decisive factors associated with abortion. Young, poorest and uneducated women were more likely to undergo unsafe abortions. Therefore, intervention studies among these target groups are warranted.

## Background

Globally a large number of women die due to birth and pregnancy-related complications and of the total, nearly 99.0% of maternal death occurs in low- and middle-income countries [1]. Abortion is one of the leading causes of maternal death. A recent study based on 115 countries in the period of 2003 to 2009 reported 7.9% of maternal deaths due to abortion [2]. The number of deaths due to abortion may be even higher, but there is a chance of under-reporting [3]. Among many factors, one of the most important contributing factor to maternal mortality in low- and middle-income countries is unsafe abortion [1]. In Nepal, abortion is reported to be the third leading cause of maternal death [4]. Abortion service was legalized in the year 2002 and services started in 2004 [5] with both public and private sectors providing surgical and medical abortion throughout the country.

Abortion may occur spontaneously or intentionally, the later also known as induced abortion, which may be either safe or unsafe. Abortion (especially unsafe) may have serious health consequences and cause complications such as hemorrhage, sepsis and uterine perforation [6, 7]. The global rate of abortion has been constant at 28–29/1000 women aged 15–44 years from 2003 to 2008, but the proportion of unsafe abortions has increased from 44.0% in 1995 to 49.0% in 2008 [8]. The rate of unsafe abortion is quite high in South-Asia (1/3 of the globe) due to strict anti-abortion legislation in many South-Asian countries [9]. Sex-selective abortion is also high in this region due to the preference for a male child [10–13].

However, many women in this region are still not aware of the legal provision for abortion and its consequences. An earlier study from Nepal reported that only 44.0% of the women were aware of the legal provision of abortion in Nepal [14]. Another study reported that most women are unaware of the availability of various abortion services in Nepal [15]. Especially young, economically deprived and those without a supportive male partner are at higher risk of unsafe abortion [16]. An earlier study reported that rich and well-educated women are more likely to have an abortion than are poor and illiterate women [17]. However, there is no clear and established evidence on this issue, especially in low- and middle-income countries. In this study, we aimed to investigate the prevalence and various factors associated with abortion and unsafe abortion using a nationally representative sample of Nepalese women. In the case of unsafe abortions, our study design aimed to examine those whose abortions were carried out by non-registered and non-trained practitioners and health workers who are not listed or certified on safe abortion care according to reformed abortion law of the Government of Nepal 2003 [18].

## Methods

### Participants and design

Demographic and Health Surveys (DHS) are nationwide household surveys conducted with the assistance of United States Agency for International Development (USAID), at about every five-year intervals in most low- and middle-income countries around the globe. This study is based on the Nepal Demographic and Health Surveys (NDHS) 2011. The study population in this study is those who responded to women’s NDHS questionnaire in 2011 and was limited to those women who ever had a terminated pregnancy (*N* = 2395). The sampling procedure, questionnaire validation and data validation of the NDHS study have been described in detail elsewhere [5]. The ethics committee of Nepal Health Research Council (NHRC) approved the study.

### Measurement of variables

#### Abortion and unsafe abortion

The abortion variable was based on a dichotomized response: *“yes”* if a woman had undergone an abortion and *“no”* if the respondent had not undergone an abortion in the last five years*.* Unsafe abortion was assessed according to the service providers who performed the abortion using a question “whom did you see to get the abortion done?” with the responses (doctor; nurse/midwife; health assistant; maternal and child health worker; village health worker; pharmacist; traditional birth attendant; female community health volunteer; relative/friend; traditional healers etc.). The responses were dichotomized to, *“safe”* if the abortion was performed by registered and trained doctors and nurses and *“unsafe”* for other than doctors and nurses depending upon who were and were not listed (certified on safe abortion care) as safe abortion service providers according to the reformed abortion law of the Government of Nepal 2003 [18].

#### Factors related to abortion

The reason for abortion was assessed using the question “What was the main reason for your most recent abortion?” From the various reasons provided in the response options, four categories were formed for the analysis namely “health risk” (health risk to mother and child), “Child spacing” (wanted a space between children), “unwanted child” (child’s sex, father did not want the child, did not want any more children) and “low earning and others” (no money to take care of the baby and others). Awareness of whether abortion is legal in Nepal was assessed using the question “Is abortion legal in Nepal?” using the response options *yes/no.* Likewise, awareness of a place for safe abortion services was based on the question “Do you know a place for safe abortion services?” with a *yes/no* response.

#### Socio-economic characteristics

The demographic variables used in this analysis were age in years (15–24, 25–34 and 35 and above), type of residence (rural, urban), educational level (no education, primary, secondary and higher), religion (Hindu, Buddhist, Muslim and others), ethnicity of the women (Brahmin and Chhetri, Janajati, Dalit and others) and ecological region (Mountain, Hill and Terai). Likewise, the wealth index of the women (Poorest, Poorer, Middle, Richer, and Richest) were also used in the study. The detailed procedures for the formation of the wealth index quintiles have been discussed elsewhere [19]. There is a wide range of ethnicities and regional identities prevailing in Nepal therefore, ethnic groups, and region of residence are used as probable determinants of the outcome. The detailed description of the caste system, superiority and inferiority among ethnic groups, diversity in regional identities plus ethnic discrimination have been described elsewhere according to NDHS 2006 [20].

### Statistical analysis

**Fig. 1:**
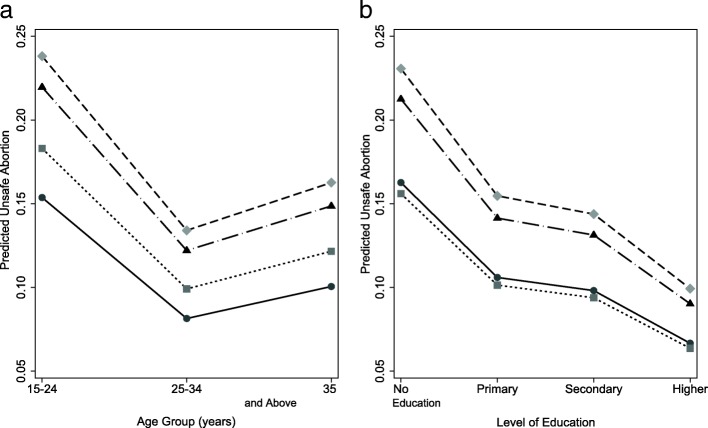
Predictive margins of the reason for abortion (**a**) by age groups and (**b**) level of education to predict unsafe abortions (*n* = 81) among Nepalese women. Circle and solid lines indicate “health risk” as a reason of abortion; Square and dotted lines indicate “unwanted child”; Triangle and dashed lines indicate “Low earning and others” and Diamond and dashed lines indicate “Child spacing”

Sampling weight was used to control for unequal selection probabilities in the data and to facilitate the generalizability of results. Descriptive characteristics of the subjects are presented as frequencies and percentages. Binary logistic regression models with robust function were used to calculate regression coefficients (Odds ratios, ORs; with their 95% confidence intervals, CIs) of abortions and unsafe abortion due to different socio-demographic characteristics of the study population. Three different models were constructed. Model І was the bivariate association of the outcomes with each of the socio-demographic variables. In Model II all variables were entered simultaneously to adjust for the effect of each of those variables. In the final model (Model ІІІ) backward stepwise elimination of the variable was applied (those with *p* > 0.05 were simultaneously removed). Thus, as post-estimation, predictive margins and their 95% CI for reason/age and reason/education to predict unsafe abortion were calculated. All analyses were performed with Statistical Package for Social Sciences (SPSS) version 21.0 for Windows (IBM Corporation, Armonk, NY, USA). The estimates for predictive margins and graph (Fig. 1) were prepared in Stata 14.0 (StataCorp LP, Texas, USA).

## Results

The prevalence of abortion and type of abortion (safe or unsafe) by demographic characteristics of the women are presented in Table 1. The five-year prevalence of abortion was 21.1% in this study population. The prevalence of abortion was higher in the age group 25–34 years (33.0%) followed by the age group 15–24 years (27.0%). According to the wealth index quintiles, the highest prevalence of abortion was reported among the richest women (27.0%) and the lowest among the poorest women (13.0%). The five-year prevalence of unsafe abortion was 16.0% out of total abortions. Unsafe abortion was higher (20.0%) in the younger age group women (15–24 years). Likewise, the prevalence of unsafe abortion was higher among the women of Muslim and other religions (19.0%) and those who were in the poorest wealth quintiles (33.0%). More than two thirds (70.0%) of the women knew about places for safe abortion services in Nepal and 44.0% of the women were aware that abortion had been legalized. Similarly, of the various reasons for performing abortions, the highest number of women (46.0%) reported unwanted child followed by low earnings and others (24.0%).

**Table 1:** Prevalence and types of abortion by demographic characteristics and knowledge of women regarding abortion

Demographic Characteristics and knowledge on abortion	*N* = 2395 n(%)	Prevalence of abortion, n(%)		Types of abortion, n(%)	
		No (1889)	Yes (506)	Safe(425)	Unsafe (81)
Age ^a^					
15–24	303(13)	222(73)	81(27)	65(80)	16(20)
25–34	940(39)	634(67)	306(33)	263(86)	43(14)
≥ 35	1152(48)	1033(90)	119(10)	97(82)	22(18)
Education					
No education	1152(48)	1019(88)	133(12)	103(77)	30(23)
Primary	451(19)	331(73)	120(27)	104(87)	16(13)
Secondary	638(27)	430(67)	208(33)	177(85)	31(15)
Higher	153(6)	108(71)	45(29)	41(91)	4(9)
Occupation					
Agriculture	1336(56)	1106(83)	230(17)	187(81)	43(19)
Unemployed	487(20)	364(75)	123(25)	105(85)	18(15)
Non-agriculture	572(24)	419(73)	153(27)	133(87)	20(13)
Religion					
Muslim and others	160(7)	133(83)	27(17)	22(81)	5(19)
Buddhist	143(6)	112(78)	31(22)	30(97)	1(3)
Hindu	2092(87)	1644(79)	448(21)	373(83)	75(17)
Ethnicity					
Dalit and others	469(19)	395(84)	74(16)	59(80)	15(20)
Janajati	786(33)	630(80)	156(20)	135(87)	21(13)
Brahmin and Chhetri	1140(48)	864(76)	276(24)	231(84)	45(16)
Region					
Mountain	354(15)	299(84)	55(16)	43(78)	12(22)
Terai	1107(46)	858(78)	249(22)	215(86)	34(14)
Hill	934(39)	732(78)	202(22)	167(83)	35(17)
Residence					
Rural	1634(68)	1315(80)	319(20)	265(83)	54(17)
Urban	761(32)	574(75)	187(25)	160(86)	27(14)
Wealth Index					
Poorest	471(20)	411(87)	60(13)	40(67)	20(33)
Poorer	383(16)	323(84)	60(16)	49(82)	11(18)
Middle	393(16)	311(79)	82(21)	66(80)	16(20)
Richer	471(20)	350(74)	121(26)	105(87)	16(13)
Richest	677(28)	494(73)	183(27)	165(90)	18(10)
Contraceptive use					
None	1121(47)	907(81)	214(19)	181(85)	33(15)
Traditional methods	208(9)	135(65)	73(35)	61(84)	12(16)
Modern methods	1066(44)	847(79)	219(21)	183(84)	36(16)
Knowledge on legal abortion					
No	1348(56)	1181(88)	167(12)	136(81)	31(19)
Yes	1047(44)	708(68)	339(32)	289(85)	50(15)
Knowledge on safe places					
No	726(30)	696(96)	30(4)	24(80)	6(20)
Yes	1669(70)	1193(71)	476(29)	401(84)	75(16)
Reason for abortion					
Health risk	69(13)	NA	69(13)	59(86)	10(14)
Child spacing	85(17)	NA	85(17)	71(84)	14(16)
Unwanted child	232(46)	NA	232(46)	196(84)	36(16)
Low earning and others	120(24)	NA	120(24)	99(82)	21(18)

*NA* Not applicable; ^a^age is categorized in 10 years interval due to few respondents lower age groups

Table 2 presents the crude and adjusted association of the demographic variables and knowledge related variables with abortion. According to the bivariate model (Model I), women of the age group 35 years and above had lower odds of abortion than women in the youngest age group. The association remained significant when variables were simultaneously added into the model (Model II) and after stepwise elimination (OR 0.37; 95% CI 0.24, 0.56). Abortion was associated with educational level in the bivariate model. The association remained significant in Model II and persisted in Model III (primary, OR 2.21; 95% CI 1.52, 3.20 and secondary, OR 1.69; 95% CI 1.22, 2.34). Similarly, women in the richest wealth quintile were more likely to have an abortion than poorer women. However, the significant association for the richer women was lost in Model II. Women of Buddhist (OR 2.15; 95% CI 1.04, 4.44) and Hindu religion (OR 1.73; 95% CI 1.00, 2.98) were more likely to undergo an abortion compared to Muslims and others. Those who knew about legal abortion were more likely to undergo abortion compared to those who did not know. Likewise, those who knew about places for safe abortion were more likely to undergo abortion.

**Table 2:** Odds ratio (OR) and their 95% confidence intervals (CIs) for abortion due to various characteristics

Demographic Characteristics and knowledge	*n* = 506^a^	Model I		Model II		Model III	
		OR	95% CI	OR	95% CI	OR	95% CI
Age							
15–24	81	1.00	Referent	1.00	Referent	1.00	Referent
25–34	306	1.37	0.96,1.94	1.31	0.89,1.91	1.37	0.94,2.00
≥ 35	119	0.30	0.21,0.45	0.33	0.21,0.51	0.37	0.24,0.56
Education							
No education	133	1.00	Referent	1.00	Referent	1.00	Referent
Primary	120	3.22	2.31,4.48	2.04	1.39,2.98	2.21	1.52,3.20
Secondary	208	4.04	2.99,5.44	1.38	0.95,2.00	1.69	1.22,2.34
Higher	45	3.79	2.37,6.06	0.92	0.52,1.65	1.25	0.78,2.05
Occupation							
Agriculture	230	1.00	Referent	1.00	Referent		
Unemployed	123	1.58	1.17,2.14	1.14	0.79,1.68		
Non-agriculture	153	1.82	1.37,2.42	1.03	0.72,1.48		
Religion							
Muslim and others	27	1.00	Referent	1.00	Referent	1.00	Referent
Buddhist	31	2.21	1.14,4.29	2.02	0.97,4.23	2.15	1.04,4.44
Hindu	448	1.83,	1.11,3.03	1.69	0.94,3.02	1.73	1.00,2.98
Ethnicity							
Dalit and others	74	1.00	Referent	1.00	Referent		
Janajati	156	1.44	0.99,2.08	1.24	0.79,1.94		
Brahmin and Chhetri	276	1.92	1.35,2.71	1.38	0.91,2.09		
Region							
Mountain	55	1.00	Referent	1.00	Referent		
Terai	249	1.45	1.02,2.04	1.10	0.72,1.69		
Hill	202	1.47	1.04,2.08	1.42	0.95,2.13		
Residence							
Rural	319	1.00	Referent	1.00	Referent		
Urban	187	1.39	1.09,1.77	0.93	0.68,1.28		
Wealth Index							
Poorest	60	1.00	Referent	1.00	Referent		
Poorer	60	1.23	0.79,1.91	0.93	0.55,1.56		
Middle	82	1.60	1.04,2.45	1.06	0.63,1.78		
Richer	121	2.13	1.42,3.19	1.17	0.70,1.96		
Richest	183	2.90	1.99,4.22	1.33	0.72,2.43		
Contraceptive use							
None	214	1.00	Referent	1.00	Referent	1.00	Referent
Traditional methods	73	2.20	1.49,3.24	1.87	1.17,3.00	1.97	1.24,3.14
Modern methods	219	0.88	0.68,1.14	0.86	0.65,1.15	0.88	0.66,1.17
Knowledge on legal abortion							
No	167	1.00	Referent	1.00	Referent	1.00	Referent
Yes	339	3.38	2.63,4.34	1.81	1.35,2.44	1.88	1.41,2.52
Knowledge on safe places for abortion							
No	30	1.00	Referent	1.00	Referent	1.00	Referent
Yes	476	7.99	4.98,12.82	5.07	3.09,8.31	4.96	3.04,8.09

*OR* Odds Ratio, *CI* Confidence Interval, Model I: crude model, Model II: simultaneously adjusted, Model III: backward stepwise elimination; ^a^n represents those who had undergone an abortion (506 out of 2395 selected respondents)

Table 3 shows the bivariate and multivariable model for unsafe abortion. In bivariate model, women in the age group 25–34 years were less likely to undergo unsafe abortion than the youngest age group. The association gained statistical significance after backward stepwise elimination in Model III (OR 0.43; 95% CI 0.19, 0.97). Women from poorer, middle, richer and the richest wealth quintile had significantly lower odds of having unsafe abortion than the poorest women in the bivariate analysis. The association remained significant in Model II and Model III (richest quintile OR 0.10; 95% CI 0.04, 0.25). Surprisingly, women residing in urban areas had higher odds of unsafe abortion (OR 2.09; 95% CI 1.06, 4.12) than those residing in rural areas. Women reporting “child spacing” as the reason for abortion had the highest odds of going through unsafe abortions as compared to those who reported health risks as the reason in all models, but none of the estimates were statistically significant.

**Table 3:** Odds ratio (OR) and their 95% confidence intervals (CIs) for unsafe abortion due to various characteristics

Demographic Characteristics and knowledge	*n* = 81^a^	Model I		Model II		Model III	
		OR	95% CI	OR	95% CI	OR	95% CI
Age							
15–24	16	1.00	Referent	1.00	Referent	1.00	Referent
25–34	43	0.51	0.24,1.09	0.49	0.21,1.11	0.43	0.19,0.97
≥ 35	22	0.71	0.30,1.69	0.66	0.25,1.77	0.52	0.22,1.20
Education							
No education	30	1.00	Referent	1.00	Referent		
Primary	16	0.64	0.30,1.41	0.84	0.36,2.00		
Secondary	31	0.59	0.31,1.14	1.35	0.58,3.63		
Higher	4	0.40	0.12,1.38	0.62	0.13,2.93		
Occupation							
Agriculture	43	1.00	Referent	1.00	Referent		
Unemployed	18	0.89	0.44,1.83	1.74	0.70,4.33		
Non-agriculture	20	0.63	0.31,1.25	1.12	0.50,2.50		
Religion							
Muslim and others	5	1.00	Referent	1.00	Referent		
Buddhist	1	0.12	0.01,1.29	0.11	0.01,1.68		
Hindu	75	1.37	0.39,4.84	1.38	0.43,4.37		
Ethnicity							
Dalit and others	15	1.00	Referent	1.00	Referent		
Janajati	21	0.61	0.25,1.47	1.07	0.42,2.72		
Brahmin and Chhetri	45	0.79	0.37,1.68	1.27	0.57,2.85		
Region							
Mountain	12	1.00	Referent	1.00	Referent		
Terai	34	0.34	0.15,0.75	0.60	0.22,1.68		
Hill	35	0.48	0.22,1.04	0.51	0.21,1.28		
Residence							
Rural	27	1.00	Referent	1.00	Referent	1.00	Referent
Urban	54	0.99	0.58,1.79	2.01	0.96,4.20	2.09	1.06,4.12
Wealth Index							
Poorest	20	1.00	Referent	1.00	Referent	1.00	Referent
Poorer	11	0.35	0.14,0.91	0.31	0.12,0.82	0.30	0.l2,0.78
Middle	16	0.34	0.14,0.86	0.24	0.10,0.61	0.30	0.12,0.73
Richer	16	0.24	0.10,0.59	0.13	0.05,0.37	0.17	0.07,0.44
Richest	18	0.15	0.06,0.33	0.06	0.02,0.20	0.10	0.04,0.25
Contraceptive use							
None	33	1.00	Referent	1.00	Referent		
Traditional methods	12	1.28	0.54,3.05	1.54	0.65,3.65		
Modern methods	36	1.04	0.57,1.90	1.13	0.60,2.12		
Knowledge on legal abortion							
No	31	1.00	Referent	1.00	Referent		
Yes	50	0.77	0.43,1.38	0.97	0.52,1.81		
Knowledge on safe places for abortion							
No	6	1.00	Referent	1.00	Referent		
Yes	75	0.56	0.19,1.65	0.96	0.36,2.55		
Reason for abortion							
Health risk	10	1.00	Referent	1.00	Referent		
Child spacing	14	1.43	0.49,4.17	2.45	0.83,7.30		
Unwanted child	36	1.00	0.41,2.49	1.46	0.59,3.63		
Low earning and others	21	1.42	0.54,3.73	1.95	0.72,5.28		

*OR* Odds Ratio, *CI* Confidence Interval, Model I: crude model, Model II: simultaneously adjusted, Model III: backward stepwise elimination; ^a^n represents those who had undergone an unsafe abortion (81 out of 506 total abortions)

The interaction of reason for undergoing abortion with age and educational status in predicting unsafe abortion was checked using a likelihood ratio test and was found statistically significant. Figure 1 presents the adjusted predicted margins of different reasons for undergoing abortion to predict unsafe abortion according to age and level of education. Younger women who reported “child spacing” as the reason for abortion had an approximately 25.0% chance of going through an unsafe abortion, which was higher than in other age groups. Likewise, uneducated women reporting “child spacing” as the reason for abortion were more likely than their peers to undergo unsafe abortion. However, those who were highly educated and belonged to the age group 25–34 years and reporting “health risks” as the reason had a less than 10.0% chance of undergoing unsafe abortion and they were safest of all. Furthermore, those who were uneducated and young aged and reported low earning as the reason of undergoing abortion had a more than 20% chance of undergoing unsafe abortion.

## Discussion

The five-year prevalence of abortion was 21.1% with 16.0% of all abortions being unsafe. Women of older age (35 years and above) were less likely to undergo both abortion and unsafe abortion. Educated women were more likely to undergo an abortion along with those who had knowledge of legal abortion and those who had knowledge of places for safe abortion services. Being rich was protective against unsafe abortion. Child spacing was the most common reason for abortion.

The prevalence of abortion is still high in our sample than in other countries with similar settings and the rate of unsafe abortion was comparable but slightly lower compared to the countries with similar settings. Higher abortion prevalence rates have been reported in Ghana with an overall prevalence of 10.0% with 45.0% of abortions being unsafe [16]. These findings are comparable despite the fact that the countries are from two different regions as both of these studies are based on nationally representative samples. However, the higher rate of unsafe abortion in the Ghanaian study can be attributed to lack of awareness among Ghanaian women regarding the law related to abortion in Ghana. A brief study on abortion in Ghana by Sedgh (2010) reported that 11.0% of maternal deaths were caused by unsafe abortions. Likewise, women were more likely to visit unsafe abortion providers due to lack of knowledge about legal abortion on different grounds, and this excludes them from post-abortion care [21]. Another study from India reported a 3.8% prevalence of abortion [22], which is reportedly lower than ours. Similarly, another study from China has reported a 22.0% prevalence of abortion [17]**,** which is more or less similar to that of our study populations.

Our study corroborates the findings of other studies, which have reported a higher rate of abortion among younger age groups [23, 24]. Earlier study from Nepal reported that the rate of medical abortion is higher in younger age groups than among older women [25]. Our study contradicts the finding of some earlier studies, which have reported older age coincides with higher rates of abortion [16, 17, 26]. We identified that younger women had a higher likelihood of having an abortion in an unsafe way than did older women, and this concurs with a study in Pakistan [27] and in Nepal [14], but contradicts a study from Ghana reporting higher odds of unsafe abortions among older women [16]. The higher odds of unsafe abortion among younger women in our study may be attributed to the lack of knowledge about safe abortion services and the age limit for legal abortion services. Similarly, our study findings suggest that the higher the education of the women the higher was the rate of abortion, which supports the evidence provided by other studies from Nepal and other low- and middle-income countries [15–17, 23, 24, 26]. The possible reason for educated women having higher rates of abortion could be their employment status and lack of time to care for children. We found higher odds of abortion among richer women, which corroborate the findings of a Ghanaian study [16]. Wealth defines the purchasing power of an individual, thus richer women have higher purchasing power than poorer women do. We found the association between wealth index and unsafe abortion, which suggests that richer women are less likely to undergo unsafe abortion than poor women do. These results corroborate with the findings of a Ghanaian study showing higher chances of safe abortion among women in the rich quintile [16].

We found no difference in the rate of abortion between urban and rural residents, which contradicts earlier findings from Nepal suggesting higher abortion rates among rural women [15]. Similarly, we found the higher rates of abortion among women in Hill and Terai than those in Mountain region which could possibly be explained by better availability and accessibility of abortion services in those regions. Unsafe abortion, however, was more likely among residents of the Mountain region, which is most socioeconomically deprived region and lacking proper abortion services. These findings are comparable to a Brazilian study reporting a higher concentration of unsafe abortion among those residing in the most socially and economically deprived regions of that country [28]. Child spacing was one of the major reason for abortion reported by the women in the younger age group and those who were uneducated. Chronological to that, child spacing as a major reason accounted for a higher prevalence of unsafe abortions in our study. We found that urban women were more likely to undergo unsafe abortion than rural women. This surprising result can be attributed to the stigma attached to abortion. In Nepal, generally, women cannot talk openly about abortion and choose for clandestine abortions that are mostly unsafe. A qualitative approach with homogenous groups could help in probing into the factors associated with the problem of cultural taboos and stigma related to abortion. A qualitative study in Mexico explored factors behind the abortion stigma and concluded that these stigmas result from the norms that place a high value on motherhood and some religious conservativeness [29]. These findings are applicable to our study as well. Qualitative studies with in-depth interviews and focus group discussions are warranted to learn more in order to design effective interventions. This study has tried to contribute to the current body of literature with scarce information in the area of abortion and risk factors in Nepal, so further studies with longitudinal design are required to delve deeper and gather additional information in this area.

The data for this study were extracted from the NDHS 2011, which is one of the largest studies conducted in Nepal using a nationally representative sample. The questionnaires used are comparable to the reference standard to measure external validity. These data are used by various international and national nongovernmental organizations in planning their interventions at different levels. The response rate (98.0%) of this study was very high compared to other national level surveys [5]**.** The chance of recall bias was addressed by including only those who ‘ever had a terminated pregnancy’. Although our data cover women having at least one birth in the last five years, we failed to include the exact time when the recent abortion took place in the period of five years preceding data collection. Only limited variables related to abortion could be studied from NDHS, as this topic was included in the survey for the first time. Access to the places providing safe abortion services could not be studied due to unavailability of the data on those institutions listed by reformed abortion law of the Government of Nepal. The cross-sectional nature of the data could be the other shortcoming of our study, as longitudinal studies are preferred to establish causal relationships between exposures and response like abortions [30]. Most of the younger, poor and uneducated women could undergo unsafe abortion due to several reasons like sex selectiveness (preference for a male child), early marriage and lack of financial resources to take care of a child. There may be underreporting of abortions, especially among rural groups, the uneducated and those who are very young age compared to those who are educated and residing in urban areas, which signifies a potential bias in the estimates of association. This bias was addressed by using the other possible factors like knowledge about legal abortion, occupation, knowledge of place for safe abortion services and use of contraceptives in the simultaneously adjusted model (model II) which could play a vital role in controlling the overestimates.

## Conclusons

The prevalence of abortion is still high in Nepal and the proportion of unsafe abortion is alarming. We found that abortion was associated with age, religion, education, and knowledge on legal abortion and a safe place to undergo abortion. Women in the poorest wealth quintile and those who had lower educational attainments and those who were younger were more likely to undergo unsafe abortion. Therefore, intervention studies among these target groups are warranted.

## Abbreviations

CI: Confidence interval
DHS: Demographic and Health Surveys
NDHS: Nepal Demographic and Health Surveys
NHRC: Nepal Health Research Council
OR: Odds ratios
USAID: United States Agency for International Development
